# Modulation of Antigen-Specific T-Cells as Immune Therapy for Chronic Infectious Diseases and Cancer

**DOI:** 10.3389/fimmu.2014.00293

**Published:** 2014-06-17

**Authors:** Suling Li, Alistair L. J. Symonds, Tizong Miao, Ian Sanderson, Ping Wang

**Affiliations:** ^1^Bioscience, Brunel University, London, UK; ^2^Blizard Institute (BICMS), Barts and the London School of Medicine and Dentistry, London, UK

**Keywords:** tolerance induction, antigen-specific T cells, bystander T-cells, nanoAPC, reverse tolerance

## Abstract

T-cell responses are induced by antigen presenting cells (APC) and signals from the microenvironment. Antigen persistence and inflammatory microenvironments in chronic infections and cancer can induce a tolerant state in T-cells resulting in hyporesponsiveness, loss of effector function, and weak biochemical signaling patterns in response to antigen stimulation. Although the mechanisms of T-cell tolerance induced in chronic infection and cancer may differ from those involved in tolerance to self-antigen, the impaired proliferation and production of IL-2 in response to antigen stimulation are hallmarks of all tolerant T cells. In this review, we will summarize the evidence that the immune responses change from non-self to “self”-like in chronic infection and cancer, and will provide an overview of strategies for re-balancing the immune response of antigen-specific T cells in chronic infection and cancer without affecting the homeostasis of the immune system.

## Introduction

T cells are essential for robust adaptive immune responses against pathogen invasion, as well as maintaining immune tolerance to self-antigens. In the tolerant state, T cells generally fail to proliferate and produce IL-2 in response to antigen stimulation (1, 2). Anergy and immune regulation are two interconnected mechanisms that maintain peripheral tolerance to self-antigens *in vivo*. In contrast to the biochemical events induced during effective responses to pathogenic antigens, in anergy the biochemical signaling pathways in T cells are only partially activated. Activation of the calcium/calcineurin/nuclear factor of activated T cells (NFAT) pathway, but not AP1 and NFκB pathways (1–3), in anergic conditions results in the expression of tolerance associated genes such as E3-ligases (1–3). This partial TCR signaling is largely due to the lack of additional signals such as costimulatory signals and activating cytokines such as IL-2, or due to direct regulation by Treg (2). Therefore, altered expression of costimulatory signals and/or activating cytokines, or defective Treg function, results in full activation of TCR signals in response to self-antigens and may induce autoimmune responses. Recent studies have uncovered hyporesponsive phenotypes with partial activation of biochemical events in virus specific T cells in chronic infectious diseases (4, 5) and models mimicking chronic infectious conditions (5, 6). These findings indicate that during chronic infection the T-cells switch from mounting robust non-self responses to a state similar to self-tolerance due to antigen persistence and/or changes in the microenvironment. Similar to the immunological milieu of chronic infection, the tumor microenvironment contains a multitude of suppressive mechanisms that allow tumors to escape immune surveillance (4, 7). Immune hyporesponsive states have been studied in many different models *in vitro* and *in vivo* and have been categorized based on the phenotypes discovered in each tolerant state (8).

This review will briefly summarize the extracellular signals that affect self-tolerance or effector function of antigen-specific T cells. We will describe the application of these signals in therapeutic intervention and focus on the recently developed nano-technologies that can reverse the tolerant state of viral specific T cells by delivering costimulatory or cytokine signals to antigen-specific T cells.

## Altered T-Cell Responses during Chronic virus Infection and Cancer

**Figure 1:**
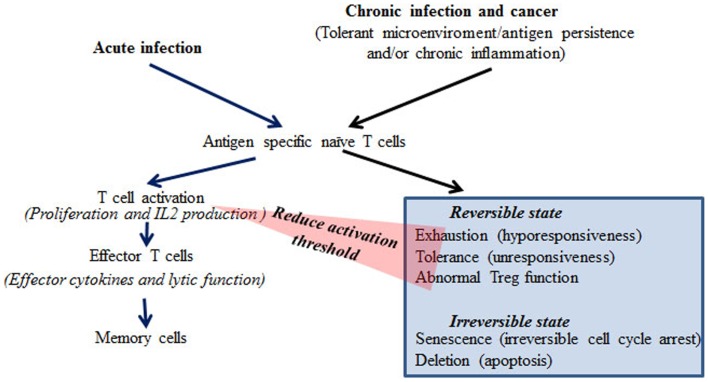
**Differential responses of T cells during acute and chronic infection or cancer**.

Chronic virus infections are associated with impaired anti-viral immunity, particularly in the infections caused by highly replicative viruses such as HIV, HBV, and HCV. In chronic infection, persistent viral antigen, and often chronic inflammation, renders T-cells dysfunctional. The mechanisms underlying dysfunctional immune responses in patients are largely unknown. Based on experimental systems studied *in vitro* and *in vivo*, different states of T-cell dysfunction have been discovered and are classified as exhaustion, tolerance, anergy, senescence, deletion, induced Treg, and ignorance based on the phenotypes, production of inhibitory cytokines such as IL-10 and TGFβ, impairment of T-cell receptor signaling molecules, and apoptosis of the T-cells in these models (Figure 1) (4–6, 8–10). These findings have been extensively reviewed (4–6, 8–10). Despite the differences in dysfunctional T-cells characterized in different model systems, the common feature is proliferative hyporesponsiveness, and impaired production of IL-2 following antigen stimulation *in vivo* or *in vitro* (4–6, 8–10). The chronic LCMV infection model resembles the observations from patients with chronic virus infections more closely than other models in terms of induction of dysfunctional T cells (4, 5). The phenotype of exhaustion of CD8 T cells in the chronic LCMV model is well-characterized, with hierarchical loss of effector cytokine production, including IL-2, TNFα, and IFNγ and impaired proliferation in response to antigen receptor stimulation *in vitro* (4, 5). In addition to this hyporesponsive phenotype, increased expression of the inhibitory costimulatory molecule PD-1 and production of the repressive cytokine IL-10 are also found in T cells from chronic LCMV infected mice (9, 10). Notably, similar phenotypes have been found in T cells from HIV, HBV, and HCV patients (11–14). Under chronic infectious conditions, viral specific CD8 T cells often lose cytotoxic function (15, 16). At the late stages of exhaustion, viral specific CD8 T cells may be deleted (5, 6). However, in contrast to CD8 T cells, viral specific CD4 T cells can persist under chronic infectious conditions, but in a hyporesponsive state (17). Therefore, there is the potential to restore CD4 responses, which may thereafter help CD8 function. It has been reported that Treg cells are increased or induced in chronic infection (18, 19). The increased Treg cells can reduce chronic inflammation from persistent viral antigen stimulation, but may also contribute to the establishment of immune tolerance toward the virus (18, 19).

Comparable to chronic infection, high levels of tumor antigens and chronic inflammation can establish an immunosuppressive microenvironment. Tumor reactive T cells have been shown to respond to tumor antigens in a similar fashion to viral specific T cells in chronic infection with expression of high levels of inhibitory costimulatory molecules such as PD-1, CTLA-4, and LAG-3 and impaired production of effector cytokines including IFNg, TNFa, and IL-2 (7, 20–22). It has been shown that advanced tumors with high loads of tumor antigens cause functional exhaustion and rapid elimination of tumor reactive T cells (23). However, in contrast to chronic viral infections, tumor antigens are generally poorly antigenic. Therefore, the frequency and avidity of tumor reactive T cells are low.

## Impaired TCR Signaling during Chronic virus Infection

We have found that antigen persistence can impair TCR signaling resulting in hyporesponsiveness (24). This hyporesponsiveness is gradually induced during antigen persistence with reduction of NFkB and AP1 activation (2, 24). This characteristic phenotype of T-cell tolerance is similar to that observed in chronic HBV infection (25). Down-regulation of TCR proximal signaling molecules has been found in CD8 T cells from chronic HBV patients (25). The impaired TCR signaling in CD8 T cells from chronic HBV patients is partly due to the down-regulation of CD3ζ (25). The reduced expression of CD3ζ is associated with up-regulation of PD-1 and impaired production of IL-2, suggesting that it is part of the mechanism leading to exhaustion (25). Viral protein Nef of HIV and E2 and core protein of HCV directly modulate TCR signaling (26). HIV Nef protein interacts with a number of TCR signaling molecules including Lyn, Hck, and Lck (27). The interaction stimulates the TCR signaling pathways in the absence of antigens leading to maintenance of viral replication (26, 27). The altered TCR signaling induced by Nef negatively affects antigen-mediated TCR signaling (28). Similarly, viral proteins from HCV also modulate TCR signaling (29). E2 protein of HCV binds CD81 and promotes TCR signaling while the core protein inhibits JNK signaling and IL-2 expression (29). However, HCV does not infect T cells. Therefore, the altered TCR responses during chronic HCV infection are largely due to the persistence of viral antigens. Whether the persistent, but abnormal, TCR signaling induced by viral proteins causes the development of T-cell exhaustion is yet to be investigated.

It has been found that the tumor microenvironment impairs the formation of T-cell immunological synapses; supramolecular structures that assemble at the T cell-APC interface (30). Dysregulated synapse formation is associated with impaired activation of Rho-GTPases and can lead to partial activation or anergy of T cells.

## Induction of Negative Costimulatory Molecules

One of the important changes to the phenotype of CD8 T cells in chronic LCMV infection is the increased expression of the negative costimulatory molecules PD-1, 2B4, CTLA-4, and LAG-3 (5). A similar phenotype of increased negative costimulatory molecules has been discovered in CD4 and CD8 T cells from chronic HBV and HIV patients (5, 15, 31–35). The function of these negative costimulatory molecules is important in the maintenance of immune tolerance toward self-antigens. Although the mechanisms underlying the induction of negative costimulatory molecules in T cells during chronic infection are not clear, it may be part of a physiological protection mechanism to reduce immunopathology induced by viral persistence and chronic inflammation. These negative costimulatory molecules are transiently up-regulated in activated effector T cells in the early stages of acute infection. However, the sustained expression of PD-1 on virus-specific CD8 T cells is associated with chronic infection, both in LCMV mouse models and in HBV patients (5). Co-expression of multiple inhibitory molecules correlates with increased functional deficits in anti-virus responses and decreased control of viral loads. Similarly, increased expression of PD-1 and CTLA-4 has been found on tumor infiltrating T cells (TIL), which can be associated with E3-ligase expression and increased Treg cells (7). Thus, the overexpression of inhibitory molecules results in shifting the balance of the immune responses from effective anti-virus or -cancer responses toward tolerance.

## Altered Cytokine Production in T Cells

One of the most pronounced changes in T cells in chronic infectious conditions is the altered production of cytokines (4–6). In contrast to acute infection, antigen-specific T cells from chronic infectious diseases fail to produce IL-2 and TNFα, but express the regulatory cytokine IL-10 (4–6). We have discovered that antigen-specific CD4 T cells gradually alter their cytokine profile in response to antigen persistence *in vivo* (24). Initial antigen stimulation effectively induces IL-2 production in antigen-specific CD4 T cells *in vivo*, while repeated exposure to the same antigen yields CD4 T cells that produce both IL-2 and IL-10 (24). Antigen persistence can finally switch off the expression of IL-2 in T cells, but these cells still produce high levels of IL-10 (24). This altered cytokine profile is associated with impaired proliferative responses and reduced AP1 and NFκB activation in response to antigen stimulation *in vivo* (24). Impaired production of effector cytokines such as IL-2, TNFα, and IFNγ is also associated with the defective activation of TCR signaling pathways and effector function of viral-specific CD4 and CD8 T cells in chronic HBV, HCV, and HIV infections (5, 6, 25). The up-regulation of inhibitory molecules, especially PD-1, is closely associated with the production of IL-10 and/or TGFβ (5, 6). Thus, virus persistence skews the T-cell response from activation and differentiation into effector cells toward antigen-specific immune tolerance. However, the mechanisms whereby IL-10 and/or TGFβ result in tolerance in chronic infections are still undefined. In the LCMV model, the lack of IL-10 or a defect in IL-10 signaling improves CD8 T-cell responses and drastically enhances the control of the infection (36, 37). TIL also display an altered cytokine profile, which is similar to that seen in chronic infections. High levels of IL-10 producing Treg cells have been found in TILs, which is associated with impaired production of IL-2, TNFa and IFNg (7, 38).

## Therapeutic Interventions to Reverse Immune Tolerance in Chronic Infection and Cancer

Therapeutic interventions for chronic viral infection and cancer aim to counter the effects of the immunosuppressive microenvironment and skew responses toward antigenic determinants that are highly immunogenic. Various approaches have been tried to increase antigen presentation quality via immunization with selected antigenic peptides, using methods such as recombinant vaccinia vaccines, DNA vaccines, peptide vaccines, and DC vaccines, to boost the anti-viral and -tumor responses (39). So far these therapeutic vaccines have not been successful. One of the possible explanations is that the hyporesponsiveness of T cells is not due to the lack of antigens, but to aspects of the chronic disease such as antigen persistence and chronic inflammation, which increase the activation threshold of T cells in response to antigen. Therefore, to overcome the high activation threshold of antigen-specific T cells in these conditions, immune therapy has to consider the antagonizing tolerogenic environment. Thus, therapeutic vaccines in combination with targeted immune modulation have been proposed as a more effective strategy to reverse the hyporesponsive state of T cells in chronic infections and cancer. In ovarian cancer, improved anti-tumor immune responses were observed after blockade of PD-1 (40). Similarly, in the LCMV model, immunization with LCMV GP33 encoding vaccinia virus coupled with administration of anti-PD-L1 blocking antibody significantly improved viral-specific CD8 T-cell responses and reduced viral load (41). Moreover, in chronic LCMV, combined therapy with a DNA vaccine and IL-10 neutralizing antibody effectively reversed viral specific CD8 T-cell tolerance (42). Immune tolerance induced by virus persistence is due to a network with multiple suppressive components. Blockade of multiple inhibitory receptors including PD-1, LAG-3, and CTLA-4 or combined blockade of inhibitory receptors and immunosuppressive cytokines achieves greater efficacy than blockade of a single inhibitory molecule in chronic LCMV models (43, 44). Although the increased T-cell function and concomitant decrease in viral load in these interventions are transient, these data support the hypothesis that reversing immune tolerance to the virus or tumor is the key for successful immunotherapy. While blockade of PD-1 and IL-10 resulted in restoration of viral specific CD8 T-cell function in a mouse model (45), the mechanisms underlying this recovery of effector function are still unknown. As many of these interventions do not specifically target the virus- or tumor-specific T cells and these pathways are important for maintenance of peripheral tolerance, it is essential to control the balance between restoration of anti-viral or -tumor responses and prevention of autoimmune diseases (5, 7). The ideal intervention will be to specifically reverse the tolerance of viral or tumor specific T cells, while maintaining the overall self-tolerance of the immune system.

## Restoring Non-Self-Responses of Viral Specific T Cells, While Maintaining the Self-Tolerance of Bystander T Cells in Chronic Infection

The differential responses of antigen-specific T cells result from biochemical signals induced in T cells following interaction with antigen-MHC complexes, costimulatory molecules, and cytokines. When the mitogenic biochemical signals break the activation threshold, the T cell will enter into the cell cycle and produce growth cytokines such as IL-2 to promote clonal expansion. Due to the persistence of viral antigen, the chronic inflammatory environment and the increased production of inhibitory molecules, the activation threshold of viral specific T cells is increased and the T cells are unable to enter the cell cycle following antigen stimulation (5, 24). However, chronic infection normally does not induce tolerance in T cells responding to antigens other than those derived from the virus itself. Therefore, systemic intervention may reverse the tolerance of viral-specific T cells, but also break the self-tolerance of bystander T cells potentially resulting in autoimmunity (5). Therefore, the ideal strategy is to reverse tolerance via modulations that increase positive and/or dampen negative costimulatory signals thereby breaking the activation threshold and driving clonal expansion of virus responding T cells, but importantly, without affecting bystander T cells. Cytokine modified and viral antigen pulsed DCs have been used to deliver antigen and positive costimulatory signals to viral specific T cells in chronic infection (46–48). However, DCs are unstable and very heterogeneous in terms of population and function. It is therefore difficult to target and deliver additional positive signals to antigen-specific T cells (49). We found that an increase in the amount of antigen presented by activated dendritic cells (DC) cannot reverse tolerance (50, 51). Although exogenous IL-2 can effectively overcome tolerance and restore the full activation of tolerant T cells in response to antigen stimulation in animal models and HBV specific CD8 T cells, systemic administration of high doses of IL-2 not only induces severe side effects, such as cardiovascular, pulmonary, hematological, hepatic, neurological, endocrine, renal, and/or dermatological complications (52), but may also promote Treg function, which can further increase the activation threshold of antigen-specific T cells (53).

**Figure 2:**
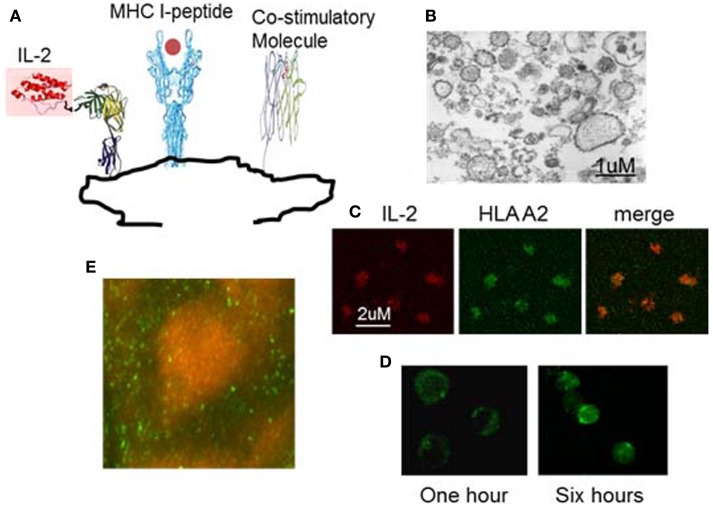
**Example of nanoAPC designed to deliver peptide-MHC complexes and IL-2 to their receptors on antigen-specific T lymphocytes**. **(A)** Graphical representation of nanoAPC. **(B)** Electron microscopy shows purified nanoAPC from IL-2 engineered 721.221 B cells. **(C)** Confocal microscopy shows nanoAPC stained with anti-IL-2 (red) and anti-HLA A2 (green). **(D)** Confocal microscopy shows interaction of nanoAPC (green) with T lymphocytes after 1 and 6 h *in vitro*. After 6 h, nanoAPCs are internalized into T cells. **(E)** Distribution of nanoAPC (green) prepared from mouse dendritic cells in T lymphocyte areas of mouse lymph node 48 h after i.v. injection. B cells were stained by B220 (orange).

In order to use IL-2 and/or anti-PD1 to overcome the hyporesponsiveness of viral specific T cells induced in chronic HBV infection while avoiding the side effects of systemic administration, we have developed a novel therapeutic vaccine (nanoAPC). These nanoAPC are derived from an APC line; the human B cell line 721.221. This cell line is MHC deficient, but expresses high levels of costimulatory molecules (51). The nanoAPC are prepared from the endoplasmic reticulum (ER) membranes of 721.221 cells (51), that are genetically engineered to express ER retained MHC class I alleles and membrane-bound IL-2. Therefore, MHC and IL-2 are synthesized physiologically in 721.221 human B cells and immobilized on ER-membranes (Figure 2) (51). After assembly with HBV antigenic peptide *in vitro*, the nanoAPC contain peptide-MHC complexes, costimulatory molecules, and IL-2. Unlike therapeutic DCs, the nanoAPC are homogeneous, stable, and can be stored at −80°C (50, 51). Equipped with defined viral-peptide-MHC complexes, the administered nanoAPC can directly interact with antigen-specific T cells *in vivo* (51). Due to the native structure of their membranes, nanoAPC effectively induce immune synapses and expression of the high affinity IL-2 receptor on T cells (Figure 2) (51). The IL-2 delivered by nanoAPC enhanced antigen-specific T-cell responses and effector function, but did not affect bystander T cells or Treg cells. When assembled with a pool of HLA A2 associated HBV peptides and HBV peptides associated with HLA DR and DP, IL-2-nanoAPC induced strong CD4 and CD8 T-cell responses in peripheral lymphocytes from chronic HBV patients (51). We demonstrated that IL-2 on nanoAPC is able to enhance TCR signaling and downregulate PD-1 expression on virus responding CD8 T cells from chronic HBV patients, which could effectively reverse tolerance as demonstrated by induction of IFNγ producing CD8 T cells in lymphocytes from chronic HBV patients (51). In addition to TCR signaling, MAPK activation can result directly from IL-2R signaling (53). It has been found that the activation of MAPK and PI3K through Shc recruited by the IL-2R is independent of STAT5 signaling in effector T cells, which differs from that in Treg cells, and is important for the expansion of activated CD8 T cells (54). We have demonstrated that nanoAPC can induce CD25 expression and immune synapse formation, which not only enables the induction of T-cell activation but also brings engineered bio-adjuvants such as IL-2 stably into signalsomes of effector T cells (51). The increased expression of CD25 on CMV antigen-specific CD8 T cells by IL-2-CMV_nlv_A2-nanoAPC is consistent with the well-known observation that IL-2 can induce CD25 expression on pre-activated CD8 T cells (51). Thus, together with peptide-MHC complexes and costimulatory molecules, the selective delivery of IL-2 is important in inducing activation of HBV responding T cells in chronic HBV patients. As the overall pharmacological dose remains low, the IL-2-nanoAPC do not activate Treg cells indicating that this approach can be adapted for use with other bio-adjuvants. Our results demonstrate that IL-2-nanoAPC, which deliver both antigen and IL-2 to antigen-responding T cells, can significantly increase functional anti-viral responses, thereby overcoming the immune tolerance induced by persistent viral load.

Nano-particles prepared from synthetic materials or genetically engineered microbes have been used to deliver antigens to DC for induction of anti-viral or -cancer immune responses (55). In contrast to these particles, nanoAPC are prepared from the ER-membranes of bio-engineered APC. Therefore, they are not only more biocompatible than synthetic nano-particles or microbes, but also deliver therapeutic molecules that are physiologically synthesized by APC seed cells. Thus, the IL-2 on IL-2-nanoAPC is more stable than free IL-2 *in vivo*, and maintains its physiological conformation allowing optimal interaction with the IL-2 receptor (data not shown). Unlike other nano-particle based vaccines, we have demonstrated that nanoAPC can directly activate T cells. NanoAPC are derived from APC cells and contain high levels of costimulatory molecules (51). Therefore, the nanoAPC mimic live DC to induce lipid raft clustering on T cells and formation of an immunological synapse, which is essential for T-cell activation. Furthermore, using HLA I negative 721.221 cells as seed cells allows us to specifically express selected HLA alleles allowing construction of HLA allele matched nanoAPC for individual patient populations.

Previously, we observed nanoAPC homing to T-cell areas of peripheral lymphoid organs, largely due to the expression of homing receptors by the cells from which the nanoAPC are derived (50). We have now further demonstrated that nanoAPC are not efficiently endocytosed by DC *in vivo* (50, 51). This is important as it allows the nanoAPC to remain as free-particles in peripheral lymphoid organs. The absence of endocytosis may be due to the lack of molecules on nanoAPC recognized by DC pattern recognition molecules (56). Thus, nanoAPC effectively target viral specific T cells and deliver immune modulation to reverse their tolerant state.

## Summary

In chronic infection and cancer, T cells are continuously confronted with moderate to high levels of antigens, which, in combination with the induced immunosuppressive microenvironment resulting from high antigen load and dysregulated immune responses, leads to increased activation thresholds and, subsequently, a reduction in effector function resulting in a tolerant state. This tolerant state can be reversed by positive regulatory molecules such as IL-2, IL-7, and/or blockade of PD-1 and CTLA-4. However, systemic administration of positive regulatory cytokines, or blocking antibodies, may cause autoimmunity. Therefore, one of the major challenges for immunotherapy against chronic infectious diseases and cancer is to reverse the tolerance of antigen-specific T cells, without affecting bystander T cells, thereby maintaining immune homeostasis to self-antigens. The development of delivery vehicles targeting antigen-specific T cells allows the provision of not only antigen but also engineered bio-adjuvant(s), which can restore effector function.

## Conflict of Interest Statement

The authors declare that the research was conducted in the absence of any commercial or financial relationships that could be construed as a potential conflict of interest.

## References

[1] Nurieva RI Liu X Dong C Molecular mechanisms of T-cell tolerance. Immunol Rev (2011) 241:133–44 10.1111/j.1600-065X.2011.01012.x 21488895 PMC5131796

[2] Schwartz RH T cell anergy. Annu Rev Immunol (2003) 21:305–34 10.1146/annurev.immunol.21.120601.141110 12471050

[3] Baine I Abe BT Macian F Regulation of T-cell tolerance by calcium/NFAT signaling. Immunol Rev (2009) 231:225–40 10.1111/j.1600-065X.2009.00817.x 19754900

[4] Kim PS Ahmed R Features of responding T cells in cancer and chronic infection. Curr Opin Immunol (2010) 22:223–30 10.1016/j.coi.2010.02.005 20207527 PMC2892208

[5] Frebel H Richter K Oxenius A How chronic viral infections impact on antigen-specific T-cell responses. Eur J Immunol (2010) 40:654–63 10.1002/eji.200940102 20077405

[6] Colonna M Viral immunosuppression: disabling the guards. J Clin Invest (2004) 113:660–2 10.1172/JCI200421166 14991061 PMC351328

[7] Nurieva R Wang J Sahoo A T-cell tolerance in cancer. Immunotherapy (2013) 5:513–31 10.2217/imt.13.33 23638746 PMC5103631

[8] Schietinger A Greenberg PD Tolerance and exhaustion: defining mechanisms of T cell dysfunction. Trends Immunol (2014) 35:51–60 10.1016/j.it.2013.10.001 24210163 PMC3946600

[9] Blackburn SD Wherry EJ IL-10, T cell exhaustion and viral persistence. Trends Microbiol (2007) 15:143–6 10.1016/j.tim.2007.02.006 17336072

[10] Wilson EB Brooks DG The role of IL-10 in regulating immunity to persistent viral infections. Curr Top Microbiol Immunol (2011) 350:39–65 10.1007/82_2010_96 20703965 PMC3492216

[11] Larsson M Shankar EM Che KF Saeidi A Ellegård R Barathan M Molecular signatures of T-cell inhibition in HIV-1 infection. Retrovirology (2013) 10:31 10.1186/1742-4690-10-31 23514593 PMC3610157

[12] Wedemeyer H He XS Nascimbeni M Davis AR Greenberg HB Hoofnagle JH Impaired effector function of hepatitis C virus-specific CD8+ T cells in chronic hepatitis C virus infection. J Immunol (2002) 169:3447–58 10.4049/jimmunol.169.6.3447 12218168

[13] Jung MC Pape GR Immunology of hepatitis B infection. Lancet Infect Dis (2002) 2:43–50 10.1016/S1473-3099(01)00172-4 11892495

[14] Guidotti LG Chisari FV Immunobiology and pathogenesis of viral hepatitis. Annu Rev Pathol (2006) 1:23–61 10.1146/annurev.pathol.1.110304.100230 18039107

[15] Day CL Kaufmann DE Kiepiela P Brown JA Moodley ES Reddy S PD-1 expression on HIV-specific T cells is associated with T-cell exhaustion and disease progression. Nature (2006) 443:350–4 10.1038/nature05115 16921384

[16] Trautmann L Janbazian L Chomont N Said EA Gimmig S Bessette B Upregulation of PD-1 expression on HIV-specific CD8+ T cells leads to reversible immune dysfunction. Nat Med (2006) 12:1198–202 16917489 10.1038/nm1482

[17] Yi J Cox M Zajac A T cell exhaustion: characteristics, causes and conversion. Immunology (2010) 129:474–81 10.1111/j.1365-2567.2010.03255.x 20201977 PMC2842494

[18] Maizels RM Smith KA Regulatory T cells in infection. Adv Immunol (2011) 112:73–136 10.1016/B978-0-12-387827-4.00003-6 22118407 PMC7150045

[19] Belkaid Y Rouse BT Natural regulatory T cells in infectious disease. Nat Immunol (2005) 6:353–60 10.1038/ni1181 15785761

[20] Mumprecht S Schürch C Schwaller J Solenthaler M Ochsenbein AF Programmed death 1 signaling on chronic myeloid leukemia-specific T cells results in T-cell exhaustion and disease progression. Blood (2009) 114:1528–36 10.1182/blood-2008-09-179697 19420358

[21] Matsuzaki J Gnjatic S Mhawech-Fauceglia P Beck A Miller A Tsuji T Tumor-infiltrating NY-ESO-1-specific CD8+ T cells are negatively regulated by LAG-3 and PD-1 in human ovarian cancer. Proc Natl Acad Sci U S A (2010) 107:7875–80 10.1073/pnas.1003345107 20385810 PMC2867907

[22] Kroemer G Galluzzi L Kepp O Zitvogel L Immunogenic cell death in cancer therapy. Annu Rev Immunol (2013) 31:51–72 10.1146/annurev-immunol-032712-100008 23157435

[23] Klein L Trautman L Psarras S Schnell S Siermann A Liblau R Visualizing the course of antigen-specific CD8 and CD4 T cell responses to a growing tumor. Eur J Immunol (2003) 33:806–14 12616501 10.1002/eji.200323800

[24] Anderson PO Sundstedt A Yazici Z Minaee S O’Neill EJ Woolf R IL-2 overcomes the unresponsiveness but fails to reverse the regulatory function of antigen-induced T regulatory cells. J Immunol (2005) 174:310–9 10.4049/jimmunol.174.8.5133-a 15611254

[25] Das A Hoare M Davies N Lopes AR Dunn C Kennedy PT Functional skewing of the global CD8 T cell population in chronic hepatitis B virus infection. J Exp Med (2008) 205:2111–24 10.1084/jem.20072076 18695005 PMC2526205

[26] Jerome K Viral modulation of T cell receptor signalling. J Virol (2008) 82:4194–204 10.1128/JVI.00059-08 18287237 PMC2293063

[27] Abraham L Fackler OT HIV-1 Nef: a multifaceted modulator of T cell receptor signaling. Cell Commun Signal (2012) 10:39 10.1186/1478-811X-10-39 23227982 PMC3534016

[28] Baur AS Sawai ET Dazin P Fantl WJ Cheng-Mayer C Peterlin BM HIV-1 Nef leads to inhibition or activation of T cells depending on its intracellular localization. Immunity (1994) 1:373–84 10.1016/1074-7613(94)90068-X 7882168

[29] Soldaini E Wack A D’Oro U Nuti S Ulivieri C Baldari CT T cell costimulation by the hepatitis C virus envelope protein E2 binding to CD81 is mediated by Lck. Eur J Immunol (2003) 33:455–64 12645944 10.1002/immu.200310021

[30] Ramsay AG Clear AJ Fatah R Gribben JG Multiple inhibitory ligands induce impaired T-cell immunologic synapse function in chronic lymphocytic leukemia that can be blocked with lenalidomide: establishing a reversible immune evasion mechanism in human cancer. Blood (2012) 120:1412–21 10.1182/blood-2012-02-411678 22547582 PMC3423779

[31] Kasprowicz V Schulze Zur Wiesch J Kuntzen T Nolan BE Longworth S Berical A High level of PD-1 expression on hepatitis C virus (HCV)-specific CD8+ and CD4+ T cells during acute HCV infection, irrespective of clinical outcome. J Virol (2008) 82:3154–60 10.1128/JVI.02474-07 18160439 PMC2258997

[32] Waggoner SN Kumar V Evolving role of 2B4/CD244 in T and NK cell responses during virus infection. Front Immunol (2012) 3:377 10.3389/fimmu.2012.00377 23248626 PMC3518765

[33] Kaufmann DE Walker BD PD-1 and CTLA-4 inhibitory cosignaling pathways in HIV infection and the potential for therapeutic intervention. J Immunol (2009) 182:5891–7 10.4049/jimmunol.0803771 19414738 PMC3726306

[34] Ahmadzadeh M Johnson LA Heemskerk B Wunderlich JR Dudley ME White DE Tumor antigen-specific CD8 T cells infiltrating the tumor express high levels of PD-1 and are functionally impaired. Blood (2009) 114:1537–44 10.1182/blood-2008-12-195792 19423728 PMC2927090

[35] Kroy DC Ciuffreda D Cooperrider JH Tomlinson M Hauck GD Aneja J Liver environment and HCV replication affect human T-cell phenotype and expression of inhibitory receptors. Gastroenterology (2014) 146:550–61 10.1053/j.gastro.2013.10.022 24148617 PMC3946973

[36] Brooks DG Trifilo MJ Edelmann KH Teyton L McGavern DB Oldstone MB Interleukin-10 determines viral clearance or persistence *in vivo*. Nat Med (2006) 12:1301–9 10.1038/nm1492 17041596 PMC2535582

[37] Ejrnaes M Filippi CM Martinic MM Ling EM Togher LM Crotty S Resolution of a chronic viral infection after interleukin-10 receptor blockade. J Exp Med (2006) 203:2461–72 10.1084/jem.20061462 17030951 PMC2118120

[38] Bergmann C Strauss L Wang Y Szczepanski MJ Lang S Johnson JT T regulatory type 1 cells in squamous cell carcinoma of the head and neck: mechanisms of suppression and expansion in advanced disease. Clin Cancer Res (2008) 14:3706–15 10.1158/1078-0432.CCR-07-5126 18559587 PMC3708468

[39] Berzofsky JA Ahlers JD Janik J Morris J Oh S Terabe M Progress on new vaccine strategies against chronic viral infections. J Clin Invest (2004) 114:450–62 10.1172/JCI22674 15314679 PMC503779

[40] Duraiswamy J Freeman GJ Coukos G Therapeutic PD-1 pathway blockade augments with other modalities of immunotherapy T-cell function to prevent immune decline in ovarian cancer. Cancer Res (2013) 73:6900–12 10.1158/0008-5472.CAN-13-1550 23975756 PMC3851914

[41] Ha SJ Mueller SN Wherry EJ Barber DL Aubert RD Sharpe AH Enhancing therapeutic vaccination by blocking PD-1-mediated inhibitory signals during chronic infection. J Exp Med (2008) 205:543–55 10.1084/jem.20071949 18332181 PMC2275378

[42] Brooks DG Lee AM Elsaesser H McGavern DB Oldstone MB IL-10 blockade facilitates DNA vaccine-induced T cell responses and enhances clearance of persistent virus infection. J Exp Med (2008) 205:533–41 10.1084/jem.20071948 18332180 PMC2275377

[43] Vezys V Penaloza-MacMaster P Barber DL Ha SJ Konieczny B Freeman GJ 4-1BB signaling synergizes with programmed death ligand 1 blockade to augment CD8 T cell responses during chronic viral infection. J Immunol (2011) 187:1634–42 10.4049/jimmunol.1100077 21742975 PMC4404506

[44] Brooks DG Ha SJ Elsaesser H Sharpe AH Freeman GJ Oldstone MB IL-10 and PD-L1 operate through distinct pathways to suppress T-cell activity during persistent viral infection. Proc Natl Acad Sci U S A (2008) 105:20428–33 10.1073/pnas.0811139106 19075244 PMC2629263

[45] Barber DL Wherry EJ Masopust D Zhu B Allison JP Sharpe AH Restoring function in exhausted CD8 T cells during chronic viral infection. Nature (2006) 439:682–7 10.1038/nature04444 16382236

[46] Akbar SM Horiike N Onji M Immune therapy including dendritic cell based therapy in chronic hepatitis B virus infection. World J Gastroenterol (2006) 12:2876–83 16718812 10.3748/wjg.v12.i18.2876 PMC4087804

[47] Zhou Y Zhang Y Yao Z Moorman JP Jia Z Dendritic cell-based immunity and vaccination against hepatitis C virus infection. Immunology (2012) 136:385–96 22486354 10.1111/j.1365-2567.2012.03590.x PMC3401977

[48] Lu W Arraes LC Ferreira WT Andrieu JM Therapeutic dendritic-cell vaccine for chronic HIV-1 infection. Nat Med (2004) 10:1359–65 10.1038/nm1147 15568033

[49] Steinman RM Hawiger D Nussenzweig MC Tolerogenic dendritic cells. Annu Rev Immunol (2003) 21:685–711 10.1146/annurev.immunol.21.120601.141040 12615891

[50] Sofra V Mansour S Liu M Gao B Primpidou E Wang P Antigen-loaded ER microsomes from APC induce potent immune responses against viral infection. Eur J Immunol (2009) 39:85–95 10.1002/eji.200838443 19089809

[51] Liu M Miao T Zhu H Symonds AL Li L Schurich A IL-2-engineered nano-APC effectively activates viral antigen-mediated T cell responses from chronic hepatitis B virus-infected patients. J Immunol (2012) 188:1534–43 10.4049/jimmunol.1102709 22210908

[52] Mier JW Aronson FR Numerof RP Vachino G Atkins MB Toxicity of immunotherapy with interleukin-2 and lymphokine activated killer cells. Pathol Immunopathol Res (1988) 7:459 10.1159/000157075 2976936

[53] Malek TR Castro I Interleukin-2 receptor signaling: at the interface of tolerance and immunity. Immunity (2010) 33:153–65 10.1016/j.immuni.2010.08.004 20732639 PMC2946796

[54] Bensinger SJ Walsh PT Zhang J Carroll M Parsons R Rathmell JC Distinct IL-2 receptor signaling pattern in CD4+CD25+ regulatory T cells. J Immunol (2004) 172:5287–96 10.4049/jimmunol.172.9.5287 15100267 PMC2842445

[55] Nandedkar TD Nanovaccines: recent developments in vaccination. J Biosci (2009) 34:995–1003 10.1007/s12038-009-0114-3 20093753

[56] Geijtenbeek TB van Vliet SJ Engering BA Hart A van Kooyk Y Self- and nonself-recognition by C-type lectins on dendritic cells. Annu Rev Immunol (2004) 22:33–54 10.1146/annurev.immunol.22.012703.104558 15032573

